# Lentinan Reduces Transmission Efficiency of COVID-19 by Changing Aerodynamic Characteristic of Exhaled SARS-CoV-2 Aerosols in Golden Hamsters

**DOI:** 10.3390/microorganisms13030597

**Published:** 2025-03-05

**Authors:** Cheng Zhang, Jiaming Li, Huan Cui, Yifei Jin, Zhaoliang Chen, Lei Zhang, Sihui Song, Bing Lu, Zhongyi Wang, Zhendong Guo

**Affiliations:** 1 Changchun Veterinary Research Institute, Chinese Academy of Agricultural Sciences, 573 Tulip Street, Changchun 130122, China; zc1349@foxmail.com (C.Z.); cuihuan1349@163.com (H.C.); zl981429835@163.com (Z.C.); 15532962603@163.com (L.Z.); songsi1029@163.com (S.S.); 2 State Key Laboratory of Pathogen and Biosecurity, Key Laboratory of Jilin Province for Zoonosis Prevention and Control, 573 Tulip Street, Changchun 130122, China; 3 College of Veterinary Medicine, Hebei Agricultural University, 2596 Lucky South Street, Baoding 071000, China; 4 Beijing Institute of Biotechnology, 20 Dongdajie Road, Beijing 100071, China; lijiaming5599@163.com (J.L.); christina_jyf@foxmail.com (Y.J.); 13693506666@163.com (B.L.)

**Keywords:** lentinan, aerosol transmissibility, exhaled virus aerosols, aerodynamic diameters

## Abstract

Lentinan (LNT) was found to reduce the aerosol transmission rate between golden hamsters from 100% (9/9) to 44.4% (4/9). The viral loads in the respiratory system, including the nasal turbinate, trachea, and lung, were significantly reduced in the infected golden hamsters that received LNT treatment. Furthermore, the amount of exhaled virus aerosols in hamsters treated with LNT was significantly lower than that in untreated hamsters throughout the entire disease progression. In detail, the amounts of virus-laden particles with aerodynamic diameters less than 5 µm exhibited a significant decreasing trend following LNT treatment. Moreover, the detection rate of infectious SARS-CoV-2 in each stage of the Anderson-6 sampler exhibited a decreasing trend following LNT treatment post-infection. In summary, our findings indicate that LNT therapy represents a promising therapeutic candidate for the treatment of COVID-19 patients. Meanwhile, during the course of treatment, LNT has the potential to reduce viral infectivity in affected individuals.

## 1. Introduction

Severe acute respiratory syndrome coronavirus 2 (SARS-CoV-2) can spread via the aerosol route, potentially accelerating its rapid global dissemination [1,2]. Previous studies have found that, during the early and middle stages following the onset of the coronavirus disease 2019 (COVID-19) symptoms, patients can exhale up to ten million virus particles per hour [3]. Furthermore, this research indicated that individuals infected with the Omicron variant are capable of releasing viral aerosols for up to two weeks post-symptom onset, without a significant reduction in the number of exhaled virus particles during breathing [3]. Furthermore, during the evolution of SARS-CoV-2, both the viral load in the upper respiratory tract and the positive rate of exhaled breath condensate were higher in patients infected with the Omicron BA.2 subvariant compared to those infected with the BA.1 subvariant [4]. Additionally, the upper limit of exhaled virus concentrations was also higher in BA.2 patients than in BA.1 patients [4]. Prolonged exhalation of high concentrations of viral aerosols can significantly increase the risk of viral transmission. Therefore, it is imperative to develop effective agents capable of inhibiting the emission of SARS-CoV-2 aerosols from COVID-19 patients.

Lentinan (LNT), a β-1,3-glucan extracted from *Lentinus edodes*, exhibits a range of pharmacological activities including immune regulation, antioxidation, anti-inflammatory, antimicrobial, and metabolic regulation, and its safety has also been affirmed [5,6]. LNT can induce immune responses by activating various immune cells and has the potential to protect the host from oxidative damage without significant adverse effects [7,8]. Consequently, LNT is commonly referred to as a “biological response modifiers” [7] and is widely utilized in the adjuvant treatment of multiple types of cancer [9,10]. Research has demonstrated that LNT also exhibits antiviral properties. Cui et al. reported that LNT mitigates acute lung injury and reduces viral loads in influenza A virus-infected mice by inhibiting the inflammatory response via TLR4/MyD88 signaling pathway [11]. LNT also represented significant antiviral activity against infectious hematopoietic necrosis virus [12], human immunodeficiency virus [13], and herpes simplex virus type 1 [14] by modulating innate immune responses and specific immunity. Fan et al. found that LNT nasal drops can shorten the time of virus clearance in COVID-19 patients [15]. In this study, we further investigated the therapeutic efficacy of LNT for SARS-CoV-2 infection and its impact on viral transmission.

The Syrian golden hamster (*Mesocricetus auratus*, Linnaeus 1758) serves as a highly susceptible animal model for investigating the pathogenesis and transmissibility of SARS-CoV-2 [16,17], as well as for evaluating therapeutic interventions such as vaccines, immunotherapies, and antiviral agents [18,19,20,21,22]. SARS-CoV-2 replicates efficiently in the respiratory tracts of hamsters and can be transmitted between them via the aerosol route. Microcomputed tomographic imaging indicated that the pulmonary injury patterns in infected hamsters closely resembled those observed in human COVID-19 cases [18]. Additionally, their compact size, cost-effectiveness, and adaptability to controlled environments make them particularly suitable for high-throughput studies requiring statistically significant sample sizes.

## 2. Materials and Methods

### 2.1. Ethics Statement

The experimental protocols involving animals were approved by the Animal Care and Use Committee of the Changchun Veterinary Research Institute, Chinese Academy of Agricultural Sciences (approval number: SMKX-20220915-16). All experiments with the SARS-CoV-2 were performed in an animal biosecurity level 3 laboratory at the Changchun Veterinary Research Institute.

### 2.2. Viruses and Cells

SARS-CoV-2 B.1.351 variant (CSTR: 16698.06.NPRC2.062100001) was propagated and titrated in African green monkey kidney epithelial cells (Vero E6) (CRL1586, ATCC, USA) [23]. The cell line was maintained in high-glucose Dulbecco’s Modified Eagle’s Medium (DMEM; Invitrogen, Carlsbad, CA, USA) supplemented with 10% fetal bovine serum (FBS; Gibco, Auckland, New Zealand), containing 100 U/mL penicillin and 100 μg/mL streptomycin. The cells were incubated at 37 °C in a humidified incubator containing 5% CO_2_. Viral titers were determined using a standard tissue culture infective dose 50% (TCID_50_) assay.

### 2.3. Hamsters

Six-week-old male Syrian golden hamsters were used in this study. They were purchased from Beijing Vital River Laboratory Animal Technology Co., Ltd. (Beijing, China) and were randomly assigned to each experimental group.

### 2.4. Source and Dosage of Lentinan

Lentinan (LNT) was purchased from Solarbio Science & Technology Co., Ltd. (Beijing, China). Lentinan dissolved in phosphate buffer solution (PBS) was given to the LNT-treated group by oral gavage at 20 mg/kg. Starting at 1-day post-infection (dpi), the golden hamsters were orally gavaged with LNT (20 mg/kg) or PBS once a day for five consecutive days.

### 2.5. Viral Load and Tissue Distribution of SARS-CoV-2 in the Respiratory Tract of Hamsters

A total of 60 hamsters were randomly divided into two groups, each comprising 30 hamsters: a LNT-treated group and a non-LNT-treated control group. Hamsters in both groups were intranasally (i.n.) inoculated with 10^5.0^ TCID_50_ of SARS-CoV-2. At 1 dpi, hamsters were orally gavaged with LNT (20 mg/kg) or PBS for five consecutive days. Nasal washes of six hamsters per group were collected at 1, 2, 4, 6, and 8 dpi, followed by euthanasia of these animals. Respiratory tissues, including lungs, nasal turbinates, and trachea, were harvested from these hamsters to determine viral titers. The tissue samples were homogenized in 1 mL of PBS using a Tissue Lyser (QIAGEN, Hilden, Germany) and subsequently centrifuged at 12,000 rpm for 10 min at 4 °C. The supernatants were then collected and titrated into Vero E6 cells. Nasal washes were directly titrated into Vero E6 cells to monitor viral shedding. Following 72 h of incubation at 37 °C, viral cytopathic effects (CPEs) were observed, and the TCID_50_ was determined by the Reed–Muench method.

### 2.6. Collection of Exhaled Viral Aerosols from Hamsters

Six hamsters were randomly divided into two groups, each comprising three hamsters: a LNT-treated group and a non-LNT-treated control group. Hamsters in both groups were i.n. inoculated with 10^5.0^ TCID_50_ of SARS-CoV-2. Starting at 1 dpi, the hamsters were orally gavaged with LNT (20 mg/kg) or PBS for five consecutive days. Exhaled aerosols were collected in accordance with our previous study [24]. An Andersen-6 sampler (TE-20-800, TISCH, Cleves, OH, USA) equipped with presterilized gelatin filters (Sartorius, Goettingen, Germany) was employed to collect exhaled aerosol samples from different groups of hamsters at a flow rate of 28.3 L/min for 1 h at 2, 4, 6, and 8 dpi. The Andersen-6 sampler fractionated the aerosol particles based on aerodynamic diameters as follows: 0.65–1.1 µm, 1.1–2.1 µm, 2.1–3.3 µm, 3.3–4.7 µm, 4.7–7.0 µm, and ≥7.0 µm. The sampler was thoroughly disinfected with 75% alcohol and dried before each sampling. Each collected filter was equally divided into two pieces: one piece was used for RNA extraction and quantification of viral copies, while the other piece was directly inoculated into Vero E6 cells to determine the presence of infectious virus in the aerosols. The experiment was independently replicated three times.

### 2.7. SARS-CoV-2 Airborne Transmission Studies in Hamsters

To investigate the effects of LNT on the airborne transmission of SARS-CoV-2, an LNT-treated group and a non-LNT-treated control group were evaluated. Nine donor hamsters per group were i.n. inoculated with 10^5.0^ TCID_50_ of SARS-CoV-2. At 1 dpi, nine naïve recipient hamsters per group were paired with the nine inoculated donors. Each recipient was housed in a new wire-frame cage adjacent to the cage housing its respective donor. The distance between the donors and recipients was 5 cm, which avoided the direct contact between the two groups of animals [25]. Starting at 1 dpi, donor hamsters were orally gavaged with LNT (20 mg/kg) or PBS for five consecutive days. To monitor viral shedding, nasal washes were collected and titrated from all animals at the indicated time points. Nasal washes were directly inoculated into Vero E6 cells. Following 72 h of incubation at 37 °C, viral CPEs were observed, and the TCID_50_ was determined by the Reed–Muench method. Serum samples were collected at 21 dpi. Then, 100 μL of virus (100 TCID_50_) was incubated with 50 μL of two-fold serial diluted sera in 96-well plates for 1 h at room temperature. Subsequently, 50 μL of Vero E6 cell suspension was added to the mixture. The cells were further incubated for 3 days at 37 °C. Viral CPEs were monitored using an inverted microscope (Olympus, Tokyo, Japan), and virus neutralization titers were determined as the reciprocal of the highest serum dilution that completely inhibited CPEs.

### 2.8. Viral Nucleic Acid Testing

RNA was extracted using the QIAamp Viral RNA Mini Kit (Qiagen, Germantown, MD, USA) and detected using the One Step PrimeScript^TM^ RT-PCR Kit (TaKaRa, Shiga, Japan) according to the manufacturer’s protocol. Quantitative real-time PCR (qRT-PCR) assays targeting regions of the *N* gene were performed using the following primers and probe: Forward primer: 5′-GGGGAACTTCTCCTGCTAGAAT-3′; Reverse primer: 5′-CAGACATTTTGCTCTCAAGCTG-3′; Probe: FAM-5′-TTGCTGCTGCTTGACAGATT-3′-TAMRA. The qRT-PCR reactions were conducted on the ABI 7500 System (ThermoFisher, Waltham, MA, USA).

### 2.9. Statistical Analysis

Quantitative data were analyzed using GraphPad Prism 5.0 software (San Diego, CA, USA) via one-way analysis of variance (ANOVA) method, and statistical comparisons between two groups were conducted using the Student–Newman–Keuls (SNK) method. All assays were performed in triplicate and are representative of at least three independent experiments. Error bars indicate the standard deviation. *p*-values less than 0.05 indicated significant differences.

## 3. Results and Discussion

**Figure 1:**
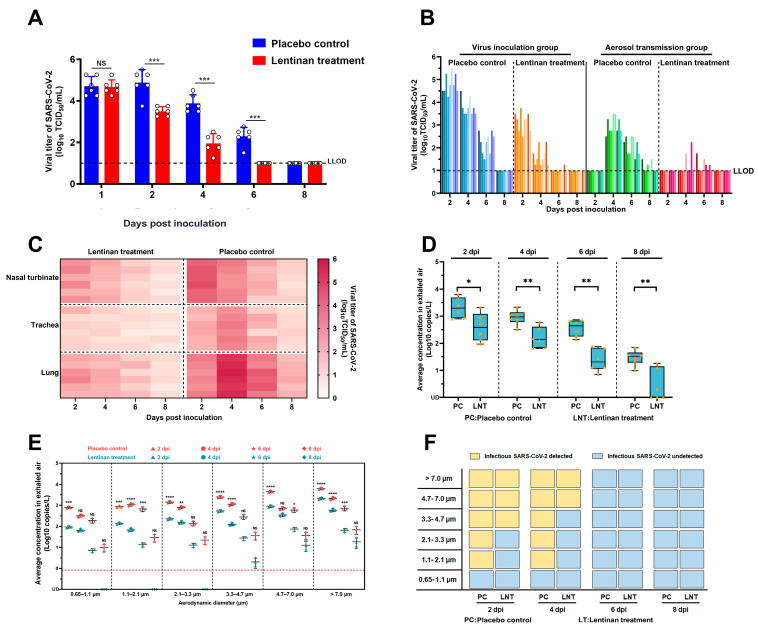
Changes in viral loads in the upper respiratory tract, viral tissue distribution, and exhaled viral aerosol amounts in golden hamsters infected with SARS-CoV-2 following LNT treatment: (**A**) Viral titers in nasal washes of infected hamsters with or without LNT treatment. The lower limit of detection (LLOD) is indicated by a black dotted line. Results are presented as the mean ± SD. All data were analyzed using one–way ANOVA method and the comparisons between two groups were performed using SNK method. NS indicates no significant difference, *** *p* < 0.001; (**B**) Viral titers in nasal washes of golden hamsters in the inoculation group and aerosol transmission group, with or without LNT treatment. Different shades of color bars within each group represent individual animals, and the LLOD is indicated with a black dotted line; (**C**) Viral titers in the respiratory tissues of golden hamsters inoculated with SARS-CoV-2 at 2, 4, 6, and 8 days post–infection (dpi), with or without LNT treatment. Darker colors represent higher viral titers; (**D**) Average concentration of exhaled viral aerosols in golden hamsters with or without LNT treatment at 2, 4, 6, and 8 dpi. Orange dots represent data from each stage of the Andersen–6 sampler. Horizontal lines indicate the maximum value, 25% percentile, 50% percentile (median), 75% percentile, and minimum value (from bottom to top), respectively. Values are expressed as the mean ± standard error of the mean (SEM). All data were analyzed using one–way ANOVA method and the comparisons between two groups were made using SNK method; * *p* < 0.05, and ** *p* <0.01; (**E**) Concentrations and sizes of viral aerosols exhaled by SARS-CoV-2–infected golden hamsters at 2, 4, 6, and 8 dpi. Pink represents the control group, while blue represents the LNT treatment group. The limit of linear range of quantification (0.833 *N* gene copies per liter of air) is indicated by the red dotted line. Values are expressed as the mean ± standard error of the mean (SEM). All data were analyzed using one–way ANOVA method and the comparisons between two groups were made using SNK method; NS means no significance, * *p* < 0.05, ** *p* <0.01, *** *p* < 0.001, and **** *p* < 0.0001; (**F**) Detection of infectious SARS-CoV-2 in each stage of Anderson–6 sampler. Yellow boxes indicate the presence of infectious SARS-CoV-2, while blue boxes indicate the absence of infectious SARS-CoV-2.

As shown in Figure 1A, there was no significant difference in nasal wash viral titers between the LNT-treated and non-LNT-treated control groups at 1 dpi, indicating that both groups of hamsters were equally well infected. Significant differences in nasal viral shedding between the two groups were observed at 2, 4, and 6 dpi. LNT treatment significantly reduced the viral shedding dose in the nasal cavity and shortened the duration of viral shedding from approximately 6 days to approximately 4 days (Figure 1A). Additionally, LNT treatment reduced the aerosol transmission rate from 100% (9/9) to 44.4% (4/9) (Figure 1B). Seroconversion results were consistent with the viral shedding data in each group (Supplemental Figure S1). These findings demonstrate that LNT treatment not only decreases SARS-CoV-2 shedding post-infection but also reduces aerosol transmissibility between hamsters. We subsequently investigated the changing trends of viral loads in the respiratory system and the aerodynamic characteristics of exhaled virus aerosols. As shown in Figure 1C, viral loads in the respiratory system, including nasal turbinates, trachea, and lungs, were significantly lower in LNT-treated infected hamsters compared to placebo control. Notably, in the placebo control group, viral loads in the trachea and lungs exhibited an increasing trend from 2 to 4 dpi, followed by a decreasing trend from 4 to 8 dpi. In contrast, LNT-treated hamsters showed a consistent decreasing trend in viral loads from 2 to 8 dpi, suggesting that LNT may inhibit viral proliferation in the trachea and lungs. Furthermore, the amount of exhaled virus aerosols in LNT-treated hamsters was significantly lower than that in untreated hamsters throughout the entire disease progression (Figure 1D). Specifically, the amounts of virus-laden particles with aerodynamic diameters less than 5 µm, including those in the size ranges of 0.65–1.1 µm, 1.1–2.1 µm, 2.1–3.3 µm, and 3.3–4.7 µm, exhibited a significant decreasing trend following LNT treatment (Figure 1E). Moreover, the detection rate of infectious SARS-CoV-2 in each stage of the Anderson-6 sampler showed a decreasing trend post-infection in LNT-treated hamsters (Figure 1F). For the LNT treatment group, infectious virus was recovered from the first three stages (>3.3 μm) of aerosol samples at 2 dpi, and from the first two stages (>4.7 μm) at 4 dpi. In contrast, for the placebo control group, infectious virus was recovered from aerosol particles of smaller sizes (>1.1 μm) at both 2 and 4 dpi. These findings indicate that LNT treatment significantly reduced the infectivity of viral particles in the small aerosol size range.

The concentration of exhaled viral aerosols determines the viral transmissibility and infection risk among susceptible populations [26]. COVID-19 patients can exhale a significant amount of viral aerosols within the first two weeks post-infection, contributing to the high aerosol transmission efficiency of SARS-CoV-2 [3]. In this study, we found that LNT treatment significantly reduced the amount of exhaled viral aerosols, thereby decreasing the infectivity of infected animals. Previous studies have shown that the virus loads in the upper respiratory tract are correlated with the concentration of exhaled viral aerosols [4]. This study further confirmed that LNT exhibits an inhibition effect by reducing viral loads in the respiratory tracts, thereby decreasing transmission efficiency between hamsters. Additionally, this study also demonstrated that LNT treatment reduced the release of virus-laden particles with aerodynamic diameters less than 5 µm, which is critical for determining whether the virus could be transmitted via the aerosol route.

The mechanism by which LNT exerts its antiviral effects against SARS-CoV-2 is multifaceted and intricate. Murphy et al. demonstrated that LNT can reduce the expression of pro-inflammatory cytokines (TNF-α, IL-8, IL-2, IL-6, IL-22, TGF-β, and IL-10), inhibit cytokine-induced NF-κB activation, and mitigate oxidative stress-induced apoptosis in vitro models of lung injury and macrophage phagocytosis [27]. Zhou et al. constructed a prognostic model of patients with gastric cancer (GC) and COVID-19, revealing that the therapeutic effects of LNT on GC/COVID-19 primarily involve modulating several neutrophil-related biological processes, as well as the nucleotide-binding oligomerization domain-like receptor (NOD-like receptor) and IL-17 signaling pathways [28]. Recent studies have suggested that SARS-CoV-2 can infect bacteria within the gut microbiota, implying its potential role as a bacteriophage [29]. Meanwhile, LNT has been demonstrated to rebalance gut microbial flora, enhance immune function, and restore intestinal barrier integrity [30,31]. This may also represent one of the mechanisms through which LNT exerts its inhibitory effects on SARS-CoV-2. Future research efforts should focus on in-depth investigations to elucidate the specific mechanisms of action.

Our study has two limitations. First, we investigated only the therapeutic effects of LNT following viral infection and did not evaluate the efficacy of prophylactic treatment. Second, we focused exclusively on the impact of LNT on the airborne transmission capacity of SARS-CoV-2, while other transmission routes, such as contact and fecal–oral transmission, were not examined.

## 4. Conclusions

In summary, our findings indicate that LNT therapy represents a promising therapeutic candidate for the treatment of COVID-19 patients. Meanwhile, during the course of treatment, LNT appears to reduce the efficiency of aerosol transmission, potentially diminishing viral infectivity in affected individuals. However, further preclinical studies are necessary to substantiate these observations made in the hamster model.

## Data Availability

The original contributions presented in this study are included in the article. Further inquiries can be directed to the corresponding authors.

## Supplementary Materials

The following supporting information can be downloaded at: https://www.mdpi.com/article/10.3390/microorganisms13030597/s1, Supplementary Figure S1. Serum antibody titers of golden hamsters in the SARS-CoV-2 inoculation group and aerosol transmission group, with or without LNT treatment, at 21 days post-infection. Different shades of color bars within each group represent individual animals, and the dashed line indicates the lower limit of detection.

## References

[1] Guo Z.D., Wang Z.Y., Zhang S.F., Li X., Li L., Li C., Cui Y., Fu R.B., Dong Y.Z., Chi X.Y. (2020). Aerosol and Surface Distribution of Severe Acute Respiratory Syndrome Coronavirus 2 in Hospital Wards, Wuhan, China, 2020. Emerg. Infect. Dis..

[2] Morawska L., Milton D.K. (2020). It Is Time to Address Airborne Transmission of Coronavirus Disease 2019 (COVID-19). Clin. Infect. Dis..

[3] Zheng J., Wang Z., Li J., Zhang Y., Jiang L., Fu Y., Jin Y., Cheng H., Li J., Chen Z. (2022). High amounts of SARS-CoV-2 in aerosols exhaled by patients with Omicron variant infection. J. Infect..

[4] Zhang Y., Li J., Jiang L., Chen Q., Fu Y., Jin Y., Chen Z., Tang F., Zeng X., Wen H. (2022). Comparison of SARS-CoV-2 aerosol emission from patients with Omicron BA.1 or BA.2 subvariant infection. J. Infect..

[5] Mao X., Hu H., Xiao X., Chen D., Yu B., He J., Yu J., Zheng P., Luo J., Luo Y. (2019). Lentinan administration relieves gut barrier dysfunction induced by rotavirus in a weaned piglet model. Food Funct..

[6] Kuang Z., Jin T., Wu C., Zong Y., Yin P., Dong W., Lin X., You W., Zhang C., Wang L. (2021). Lentinan Attenuates Damage of the Small Intestinal Mucosa, Liver, and Lung in Mice with Gut-Origin Sepsis. J. Immunol. Res..

[7] Volman J.J., Ramakers J.D., Plat J. (2008). Dietary modulation of immune function by beta-glucans. Physiol. Behav..

[8] Ren G., Li K., Hu Y., Yu M., Qu J., Xu X. (2015). Optimization of selenizing conditions for Seleno-Lentinan and its characteristics. Int. J. Biol. Macromol..

[9] Zhang M., Zhang Y., Zhang L., Tian Q. (2019). Mushroom polysaccharide lentinan for treating different types of cancers: A review of 12 years clinical studies in China. Prog. Mol. Biol. Transl. Sci..

[10] Sun M., Zhao W., Xie Q., Zhan Y., Wu B. (2015). Lentinan reduces tumor progression by enhancing gemcitabine chemotherapy in urothelial bladder cancer. Surg. Oncol..

[11] Cui H., Zhang C., Zhang C., Cai Z., Chen L., Chen Z., Zhao K., Qiao S., Wang Y., Meng L. (2022). Anti-Influenza Effect and Mechanisms of Lentinan in an ICR Mouse Model. Front. Cell. Infect. Microbiol..

[12] Ren G., Xu L., Lu T., Yin J. (2018). Structural characterization and antiviral activity of lentinan from Lentinus edodes mycelia against infectious hematopoietic necrosis virus. Int. J. Biol. Macromol..

[13] Suzuki H., Okubo A., Yamazaki S., Suzuki K., Mitsuya H., Toda S. (1989). Inhibition of the infectivity and cytopathic effect of human immunodeficiency virus by water-soluble lignin in an extract of the culture medium of Lentinus edodes mycelia (LEM). Biochem. Biophys. Res. Commun..

[14] Sarkar S., Koga J., Whitley R.J., Chatterjee S. (1993). Antiviral effect of the extract of culture medium of Lentinus edodes mycelia on the replication of herpes simplex virus type 1. Antivir. Res..

[15] Fan W., You B., Wang X., Zheng X., Xu A., Liu Y., Peng H., Yin W., Xu M., Dong X. (2023). Safety and efficacy of lentinan nasal drops in patients infected with the variant of COVID-19: A randomized, placebo-controlled trial. Front. Pharmacol..

[16] Sia S.F., Yan L.M., Chin A.W.H., Fung K., Choy K.T., Wong A.Y.L., Kaewpreedee P., Perera R.A.P.M., Poon L.L.M., Nicholls J.M. (2020). Pathogenesis and transmission of SARS-CoV-2 in golden hamsters. Nature.

[17] Chan J.F., Zhang A.J., Yuan S., Poon V.K., Chan C.C., Lee A.C., Chan W.M., Fan Z., Tsoi H.W., Wen L. (2020). Simulation of the Clinical and Pathological Manifestations of Coronavirus Disease 2019 (COVID-19) in a Golden Syrian Hamster Model: Implications for Disease Pathogenesis and Transmissibility. Clin. Infect. Dis..

[18] Imai M., Iwatsuki-Horimoto K., Hatta M., Loeber S., Halfmann P.J., Nakajima N., Watanabe T., Ujie M., Takahashi K., Ito M. (2020). Syrian hamsters as a small animal model for SARS-CoV-2 infection and countermeasure development. Proc. Natl. Acad. Sci. USA.

[19] Yahalom-Ronen Y., Tamir H., Melamed S., Politi B., Shifman O., Achdout H., Vitner E.B., Israeli O., Milrot E., Stein D. (2020). A single dose of recombinant VSV-∆G-spike vaccine provides protection against SARS-CoV-2 challenge. Nat. Commun..

[20] Rogers T.F., Zhao F., Huang D., Beutler N., Burns A., He W.T., Limbo O., Smith C., Song G., Woehl J. (2020). Isolation of potent SARS-CoV-2 neutralizing antibodies and protection from disease in a small animal model. Science.

[21] Tortorici M.A., Beltramello M., Lempp F.A., Pinto D., Dang H.V., Rosen L.E., McCallum M., Bowen J., Minola A., Jaconi S. (2020). Ultrapotent human antibodies protect against SARS-CoV-2 challenge via multiple mechanisms. Science.

[22] Kreye J., Reincke S.M., Kornau H.C., Sánchez-Sendin E., Corman V.M., Liu H., Yuan M., Wu N.C., Zhu X., Lee C.D. (2020). A Therapeutic Non-self-reactive SARS-CoV-2 Antibody Protects from Lung Pathology in a COVID-19 Hamster Model. Cell.

[23] Zhang C., Cui H., Li E., Guo Z., Wang T., Yan F., Liu L., Li Y., Chen D., Meng K. (2022). The SARS-CoV-2 B.1.351 Variant Can Transmit in Rats But Not in Mice. Front. Immunol..

[24] Guo Z., Zhang C., Zhang C., Cui H., Chen Z., Jiang X., Wang T., Li Y., Liu J., Wan Z. (2022). SARS-CoV-2-related pangolin coronavirus exhibits similar infection characteristics to SARS-CoV-2 and direct contact transmissibility in hamsters. iScience.

[25] Jin Y., Cui H., Jiang L., Zhang C., Li J., Cheng H., Chen Z., Zheng J., Zhang Y., Fu Y. (2023). Evidence for human infection with avian influenza A(H9N2) virus via environmental transmission inside live poultry market in Xiamen, China. J. Med. Virol..

[26] Guo W., Fu Y., Jia R., Guo Z., Su C., Li J., Zhao X., Jin Y., Li P., Fan J. (2022). Visualization of the infection risk assessment of SARS-CoV-2 through aerosol and surface transmission in a negative-pressure ward. Environ. Int..

[27] Murphy E.J., Masterson C., Rezoagli E., O’Toole D., Major I., Stack G.D., Lynch M., Laffey J.G., Rowan N.J. (2020). β-Glucan extracts from the same edible shiitake mushroom Lentinus edodes produce differential in-vitro immunomodulatory and pulmonary cytoprotective effects—Implications for coronavirus disease (COVID-19) immunotherapies. Sci. Total Environ..

[28] Zhou S., Sun H. (2024). Prognostic model for gastric cancer patients with COVID-19 and network pharmacology study on treatment by lentinan. Sci. Rep..

[29] Brogna C., Brogna B., Bisaccia D.R., Lauritano F., Marino G., Montano L., Cristoni S., Prisco M., Piscopo M. (2022). Could SARS-CoV-2 Have Bacteriophage Behavior or Induce the Activity of Other Bacteriophages?. Vaccines.

[30] Ji X., Su L., Zhang P., Yue Q., Zhao C., Sun X., Li K., Liu X., Zhang S., Zhao L. (2022). Lentinan improves intestinal inflammation and gut dysbiosis in antibiotics-induced mice. Sci. Rep..

[31] Zhang X., Wang L., Khan A.I., Rehman A.U., Khinsar K.H., Xin Y. (2025). Lentinan’s effect on gut microbiota and inflammatory cytokines in 5-FU-induced mucositis mice. AMB Express.

