# Supplementary material for: Lentinan Reduces Transmission Efficiency of COVID-19 by Changing Aerodynamic Characteristic of Exhaled SARS-CoV-2 Aerosols in Golden Hamsters
Source: Microorganisms. 2025 Mar 5;13(3):597. doi: 10.3390/microorganisms13030597 (PMC11944727; doi:10.3390/microorganisms13030597)

Supplementary Materials

Title: Lentinan reduces transmission efficiency of COVID-19 by changing aerodynamic characteristic of exhaled SARS-CoV-2 aerosols in golden hamsters

Figure legends

Supplementary Figure S1. Serum antibody titers of golden hamsters in the SARS-CoV-2 inoculation group and aerosol transmission group, with or without LNT treatment, at 21 days post-infection. Different shades of color bars within each group represent individual animals, and the dashed line indicates the lower limit of detection.

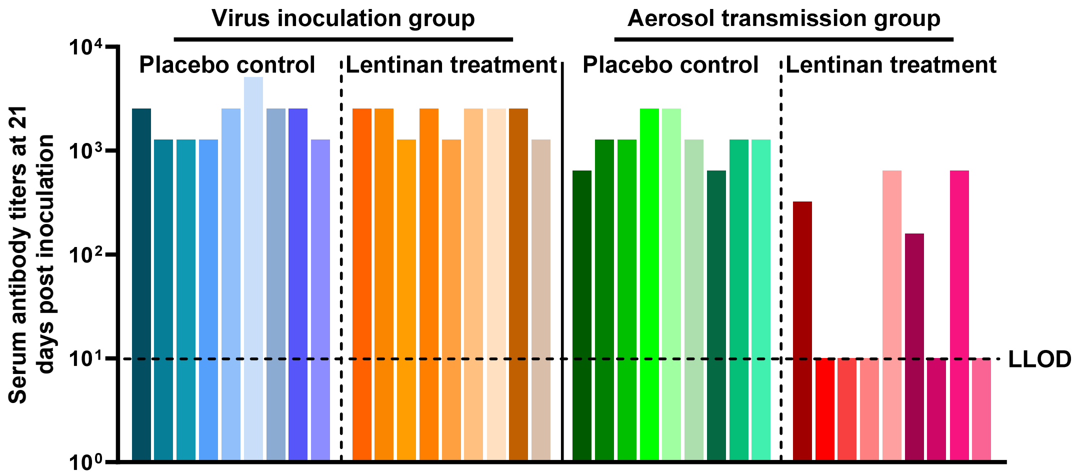

Supplement: Supplementary file 1 [file microorganisms-13-00597-s001.zip › microorganisms-3469645-supplementary.pdf]
